# Draft genome sequences of two strains of *Starmerella bacillaris* of enological interest, FC54 and MUT5705

**DOI:** 10.1128/mra.01009-25

**Published:** 2025-11-17

**Authors:** Paola Di Gianvito, Miguel Morard, Francesca Cristetti, Luca Cocolin, Kalliopi Rantsiou, Amparo Querol, Vasileios Englezos

**Affiliations:** 1 Department of Agricultural, Forest and Food Sciences, University of Turin 165514, Grugliasco, Torino, Italy; 2 Yeastomics Laboratory (SBYBI group), Food Biotechnology Department, IATA-CSIC, Valencia, Spain; 3 Interdepartmental Centre for Grapevines and Wine Sciences, University of Turin 9314, Alba, Italy; University of California Riverside, Riverside, California, USA

**Keywords:** *Starmerella bacillaris*, wine, WGS, non-*Saccharomyces*, yeast

## Abstract

*Starmerella bacillaris* is a non*-Saccharomyces* yeast associated with enological niches. We present whole-genome sequences of strains FC54 and MUT5705, previously isolated from grapes and extensively characterized for their enological traits. Given the limited genomic resources, these data provide valuable insights into the genetic diversity and heterogeneity within this species.

## ANNOUNCEMENT

*Starmerella bacillaris* (formerly *Candida zemplinina*) has gained interest for its unique enological properties (1). It is commonly found on grapes and during wine fermentation worldwide (2). Its use in mixed fermentations with *Saccharomyces cerevisiae* has been widely studied for its impact on wine composition and quality (3). This fructophilic, acidogenic, psychrotolerant, and osmotolerant yeast is well suited for sweet wine fermentations (2) and can act as a biocontrol agent against undesirable microbes (4, 5) and pests (6). During wine fermentation, most *Starm. bacillaris* strains produce less ethanol and more glycerol than *S. cerevisiae* (7–9). They can increase total acidity, while acetaldehyde, acetic acid, and pyruvic acid production is strain dependent (2, 10). This species also tolerates high ethanol levels (1).

*Starm. bacillaris* is a haploid species with no evidence of sporulation (11). Genome sequencing and karyotypic analyses demonstrated three chromosomes, an approximately 9 Mb genome (12, 13), and high genetic diversity among isolates (12). Here, we present the draft genome sequences of two *Starm. bacillaris* strains, FC54 and MUT5705, isolated from *Vitis vinifera* L. cv. Picolit grapes in the Friuli Venezia Giulia region, Italy (14). Both strains were phenotypically characterized and selected for their enological attitude in mixed fermentation with *S. cerevisiae*. MUT5705 belongs to the Mycotheca Universitatis Taurinensis MUT (Dipartimento di Scienze della Vita e Biologia dei Sistemi, University of Torino, Italy).

For whole-genome sequencing, *Starm. bacillaris* strains were grown on YPD agar medium (10 g/L yeast extract, 20 g/L peptone, and 20 g/L dextrose, and 15 g/L agar) for 3 days at 25°C, then inoculated in YPD broth and incubated at 25°C for 24 hours. High-quality genomic DNA was extracted using the Epicenter Masterpure DNA purification kit (Epicenter, Madison, WI, USA). Libraries were prepared using the Illumina DNA Prep (M) Tagmentation kit and sequenced on a NovaSeq 600 S4 flow cell (Illumina) at the University of Trento, Trento, Italy.

The *Starm. bacillaris* strains present a 99.59% similarity between the two genomes as measured by average nucleotide identity (15). The *de novo* assembly was performed using SPAdes v.4.2.0 with default parameters (16). FC54 presented a nuclear genome sequence of 9.5 Mb, while the draft genome of MUT5705 was of 9.5 Mb. FastQC v.0.11.9 was used to assess read quality. A total of 24,182,418 raw reads were generated for strain FC54. The genome assembly achieved an average depth of coverage of 371×, corresponding to a total genome coverage of 381×. For strain MUT5705, 14,992,784 reads were obtained, yielding an average depth of coverage of 235× and a genome coverage of 230×. The GC contents were 39.5% and 39.5% for FC54 and MUT5707, respectively. The high quality of the FC54 assembly was indicated by the presence of 837 scaffolds (*N*_50_ length of 769.9 kb and L50 of 6), while MUT5707 had 589 scaffolds (*N*_50_ length of 601.6 kb and L50 of 6). The completeness of the assembly was assessed using BUSCO v.5.8.2 (17), which estimated the genome sequence to be 75.4% complete for both assemblies based on full and partial targets of the Saccharomycetes_odb10 database.

## Data Availability

These Whole Genome Shotgun projects have been deposited at DDBJ/ENA/GenBank under accession number JBQWLV000000000 for MUT5705 and JBQWLW000000000 for FC54 strains, respectively. The versions described in this paper are version JBQWLV010000000 and JBQWLW010000000. The associated BioProject for both strains is PRJNA1298759, while the BioSample accession numbers are SAMN50284507 for strain FC54 and SAMN50284508 for strain MUT5705.
